# 2651. Successful Sequencing and Genotyping of SARS-CoV-2 and Influenza Viruses from Self-Collected Nasal Swabs for SARS-CoV-2 Rapid Antigen Testing

**DOI:** 10.1093/ofid/ofad500.2262

**Published:** 2023-11-27

**Authors:** Kat Schmidt, Stephanie A Richard, Emily Hone, Simon Pollett, Rezalina Tant, Michele Wayman, Chantele Friend, Kamala Thapa, Anthony C Fries, Drake Tilley, Timothy Burgess, Mark P Simons, Vivian Hogan, Rhonda Colombo

**Affiliations:** Infectious Disease Clinical Research Program, USUHS, Arlington, Virginia; Infectious Disease Clinical Research Program, Department of Preventive Medicine and Biostatistics, Uniformed Services University of the Health Sciences, Bethesda, MD, USA, Bethesda, Maryland; Infectious Disease Clinical Research Program, USUHS, Arlington, Virginia; Infectious Disease Clinical Research Program, Department of Preventive Medicine and Biostatistics, Uniformed Services University of the Health Sciences, Bethesda, MD, USA, Bethesda, Maryland; Infectious Disease Clinical Research Program, USUHS, Arlington, Virginia; Infectious Disease Clinical Research Program, USUHS, Arlington, Virginia; Infectious Disease Clinical Research Program, USUHS, Arlington, Virginia; Infectious Disease Clinical Research Program, USUHS, Arlington, Virginia; U.S. Air Force School of Aerospace Medicine, Dayton, Ohio; Naval Health Clinic Annapolis, Annapolis, MD, USA, Annapolis, Maryland; Infectious Disease Clinical Research Program, Department of Preventive Medicine and Biostatistics, Uniformed Services University of the Health Sciences, Bethesda, MD, USA, Bethesda, Maryland; Infectious Disease Clinical Research Program, Department of Preventive Medicine and Biostatistics, Uniformed Services University of the Health Sciences, Bethesda, MD, USA, Bethesda, Maryland; Infectious Disease Clinical Research Program, USUHS, Arlington, Virginia; Infectious Disease Clinical Research Program, USUHS, Arlington, Virginia

## Abstract

**Background:**

Genomic surveillance of acute respiratory infections (ARIs) is conventionally limited to nasopharyngeal PCR swab sequencing, missing many ARIs. “Acute Respiratory Infections at the Academy” (ARIA) is an augmented surveillance study of medically attended ARIs (MAARIs) at the United States Naval Academy (USNA). We leveraged this study to examine whether rapid antigen (Ag) tests performed for SARS-CoV-2 (SC2) could serve to detect and sequence SC2 and influenza viruses.

**Methods:**

ARIA began accruing data and specimens on 3/20/23 from individuals who presented to an onsite clinic with an acute illness for which a respiratory specimen (i.e., nasal or nasopharyngeal) was collected. Per standard of care (SOC), patients with suspected ARI undergo rapid Ag testing (Binax Now) for SC2 upon presentation to the clinic; if the Ag test is negative, PCR testing for SC2, influenza, and RSV is also performed. For ARIA, all swabs (including nasal swabs obtained for rapid Ag testing) are retained and sent to a partner laboratory (USAFSAM) for expanded multiplex molecular testing plus genomic sequencing of all SC2 and influenza positive specimens.

**Results:**

In the first 2 weeks of the study, 228 MAARI cases were identified, of which 14 (6.1%) were positive for SC2 or influenza by SOC rapid antigen testing (n=6) or PCR testing (n=8). Further molecular analysis at USAFSAM through multiplex PCR identified ≥ 1 pathogen in an additional 74 (32.5%) cases, most commonly rhinovirus/enterovirus (n=38) or human coronavirus NL63 (n=14) (Table 1). Among Ag swabs that had any virus detected via FluSC2 rRT-PCR testing at USAFSAM, we successfully performed viral genomic sequencing on the first 8; 1 case of influenza H1N1 clade 6B.1A.5a.2a.1 and 7 cases of SC2 variant Omicron XBB.1.5 were identified, including from one Ag swab that had low concentration of SC2 viral RNA (8.5 viral genome equivalents/reaction; PCR Ct 35.0).

**Figure:**
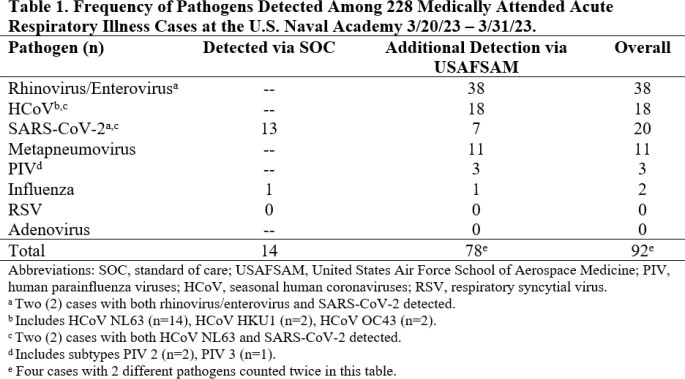

**Conclusion:**

We demonstrated the feasibility of sequencing and genotyping influenza and SC2 from a residual anterior nasal swab after use in rapid SC2 Ag testing. Further, a SC2 variant was determinable on Ag swabs with low quantities of SC2 viral RNA. This is a key proof-of-concept for ARI surveillance, especially as rapid SC2 Ag testing is increasingly used in clinical practice.

**Disclosures:**

**Simon Pollett, MBBS**, AstraZeneca: The IDCRP and the Henry M. Jackson Foundation (HJF) were funded to conduct an unrelated phase III COVID-19 monoclonal antibody immunoprophylaxis trial **Timothy Burgess, MD, MPH**, AstraZeneca: The IDCRP and the Henry M. Jackson Foundation (HJF) were funded to conduct an unrelated phase III COVID-19 monoclonal antibody immunoprophylaxis trial **Mark P. Simons, PhD**, AstraZeneca: The IDCRP and HJF were funded to conduct an unrelated phase III COVID-19 monoclonal antibody immunoprophylaxis trial as part of US Govt COVID Response

